# Assessing the Risk of Dengue Virus Local Transmission: Study on Vector Competence of Italian *Aedes albopictus*

**DOI:** 10.3390/v16020176

**Published:** 2024-01-24

**Authors:** Claudia Fortuna, Francesco Severini, Giulia Marsili, Luciano Toma, Antonello Amendola, Giulietta Venturi, Claudio Argentini, Francesca Casale, Ilaria Bernardini, Daniela Boccolini, Cristiano Fiorentini, Hapuarachchige Chanditha Hapuarachchi, Fabrizio Montarsi, Marco Di Luca

**Affiliations:** 1 National Reference Laboratory for Arboviruses, Department of Infectious Diseases, Istituto Superiore di Sanità, 00161 Rome, Italy; giulia.marsili@iss.it (G.M.); antonello.amendola@iss.it (A.A.); giulietta.venturi@iss.it (G.V.); claudio.argentini@iss.it (C.A.); cristiano.fiorentini@iss.it (C.F.); 2 Unit of Vector-Borne Diseases and International Health, Department of Infectious Diseases, Istituto Superiore di Sanità, 00161 Rome, Italy; francesco.severini@iss.it (F.S.); luciano.toma@iss.it (L.T.); francesca.casale@iss.it (F.C.); ilaria.bernardini@iss.it (I.B.); daniela.boccolini@iss.it (D.B.); marco.diluca@iss.it (M.D.L.); 3 Microbiology and Molecular Epidemiology Division, Environmental Health Institute, 11, Biopolis Way, #06-05-08, Singapore 138667, Singapore; chanditha_hapuarachchi@nea.gov.sg; 4 Istituto Zooprofilattico Sperimentale delle Venezie, 35020 Legnaro, Italy; fmontarsi@izsvenezie.it

**Keywords:** dengue virus, mosquito, arboviruses, Italy, *Aedes albopictus*, experimental infection

## Abstract

The frequency of locally transmitted dengue virus (DENV) infections has increased in Europe in recent years, facilitated by the invasive mosquito species *Aedes albopictus*, which is well established in a large area of Europe. In Italy, the first indigenous dengue outbreak was reported in August 2020 with 11 locally acquired cases in the Veneto region (northeast Italy), caused by a DENV-1 viral strain closely related to a previously described strain circulating in Singapore and China. In this study, we evaluated the vector competence of two Italian populations of *Ae. albopictus* compared to an *Ae. aegypti* lab colony. We performed experimental infections using a DENV-1 strain that is phylogenetically close to the strain responsible for the 2020 Italian autochthonous outbreak. Our results showed that local *Ae. albopictus* is susceptible to infection and is able to transmit the virus, confirming the relevant risk of possible outbreaks starting from an imported case.

## 1. Introduction

Dengue fever is a mosquito-borne tropical disease caused by four distinct but closely related serotypes of Dengue virus (DENV; family *Flaviviridae*, genus Flavivirus) that are transmitted by *Aedes* mosquitoes [1]. The species *Aedes aegypti*, widely spread in endemic areas, is considered the main vector of DENV. Although considered a secondary vector of DENV, *Ae. albopictus* is however associated with virus transmission in several areas of the world, including Europe [2]. The first Italian indigenous DENV outbreak was reported in Montecchio Maggiore (Vicenza province, Veneto region) in August 2020 with 11 cases secondary to an imported case from Indonesia [3]. To evaluate the role of *Ae. albopictus* in DENV transmission, we analyzed the vector competence, through experimental infections, of two Italian populations of *Ae. albopictus*. Potential vertical (transovarial) transmission of DENV was also evaluated. Since it was not possible to isolate the DENV-1 strain responsible for the 2020 Italian outbreak, either from mosquito pools or from human sera, for the experimental infections, we used a phylogenetically highly related DENV-1 strain circulating in Singapore.

## 2. Materials and Methods

### 2.1. Virus and Mosquito Populations

DENV-1 isolate SG (EHI)D1/30889Y14 (accession number MG097876) was selected based on the high genetic homology of 98.27% with the DENV-1 strain of the 2020 Italian autochthonous outbreak (namely VI/Italy/2020, accession number MZ291446; Supplementary File). The dengue strain was kindly provided by the Environmental Health Institute, National Environment Agency, Singapore for the purposes of the study. The virus was grown in VERO cells and titrated via plaque assay [4]. Two geographically different *Ae. albopictus* populations were collected from Rome (Lazio region, Central Italy) and Montecchio Maggiore (Veneto region, northeast Italy), the latter being the town where the 2020 DENV-1 outbreak occurred. A long-established *Ae. aegypti* laboratory colony (collected in Reynosa, Mexico, in 1998) was used as the reference. The eggs of two *Ae. albopictus* populations were collected by using ovitraps and were reared in the insectarium of the Istituto Superiore di Sanità in Rome. Adults were maintained before the test for a few generations (F3–F5) in climatic chambers under the following conditions: 26 ± 1 °C temperature; 70% relative humidity (RH); and a 14:10 h light/dark photoperiod. To check that the two *Ae. albopictus* populations were virus free, 5 pools of 20 specimens (males and females) per pool for each F0 offspring generation were analyzed for DENV via real-time PCR (qRT-PCR) [5].

### 2.2. Genetic Similarity Analysis

The sequence of the DENV-1 strain of the 2020 Italian autochthonous outbreak (VI/Italy/2020; accession number MZ291446) [3] was compared to DENV-1 sequences available in a sequence repository by using the BLASTN tool in order to identify the most related viral strains. We found more than 200 isolates showing high homology with MZ291446 (<98% homology). Few of these were cultured. MG097876, circulating in Singapore where *Ae. albopictus* was described, was selected because it was highly related (98.27%) and isolated in cells.

### 2.3. Experimental Infection

The experimental infections were performed in a biosafety level 3 laboratory with 5–10 days old female mosquitoes that were allowed to feed for 60 min using a membrane feeding apparatus containing a blood–DENV mixture. The virus was diluted in rabbit blood to a final virus concentration of 5 log_10_ plaque-forming units (PFU)/mL. The infectious blood was maintained at 37 °C through a warm water circulation system. Unfed and partially fed mosquitoes were excluded from the study; only completely engorged females were transferred to a climate chamber (set to the same environmental conditions as previously described) and were provided with a 10% sucrose solution. They were monitored for 28 days. At 0, 7, 14, 21, and 28 days post-infectious blood meal, 10–23 mosquitoes of each species and population were individually processed. To determine the vector competence, the whole body (head, thorax, and abdomen), legs plus wings, and saliva of the mosquitoes were screened for DENV RNA to estimate the infection, dissemination, and transmission rates (IR, DR, and TR), respectively [5]. Mosquito saliva was collected by inserting the entire proboscis into a single quartz capillary filled with 1 μL of Vaseline oil. Vaseline enables the clear identification of saliva droplets, helping to rule out the possibility that a negative result for the virus in a saliva sample is due to a lack of saliva production. One microliter of 1% pilocarpine, a saliva stimulant that is an analogue of acetylcholine [6], prepared in phosphate-buffered saline (PBS) at 0.1% Tween 80, was applied on the mosquito thorax. After 30 min, the medium containing the saliva was expelled under pressure from the capillary into a 1.5 mL tube containing 500 μL of mosquito diluent consisting of PBS, 20% heat-inactivated FBS, and a 1% penicillin/streptomycin/amphotericin B mix (Invitrogen Corp., Carlsbad, CA, USA; GIBCO Brl, Rockville, MD, USA). Virus titers were quantified by using crossing point values obtained from a qRT-PCR [7] and comparing them with a standard curve obtained from 10-fold serial dilutions of virus stock of known concentration [8,9]. Potential vertical transmission of DENV was also analyzed. For this, mosquitoes were allowed to lay eggs (first gonotrophic cycle—FGC) after the infectious blood meal. Larvae from the FGC were reared up to adulthood in the climatic chamber, and adults were tested for DENV RNA via qRT-PCR analysis on pools (5 pools for *Ae. albopictus* from Rome, 3 pools for *Ae. albopictus* from Montecchio Maggiore, and 4 pools for *Ae. aegypti*), consisting of 5 mosquitoes/pool.

### 2.4. Data Analysis and Statistics

Statistical significance tests were performed using a parametric Student’s *t* test. All statistical analyses were performed using GraphPad Prism 5 software (GraphPad Software, San Diego, CA, USA). For all analyses, a *p*-value ≤ 0.05 was considered significant.

## 3. Results

**Figure 1:**
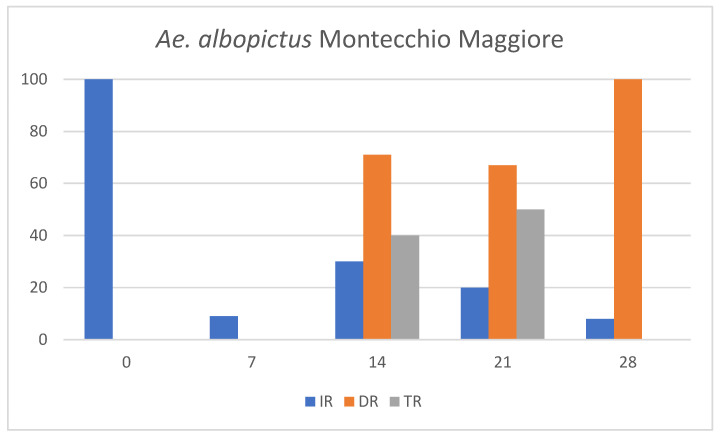

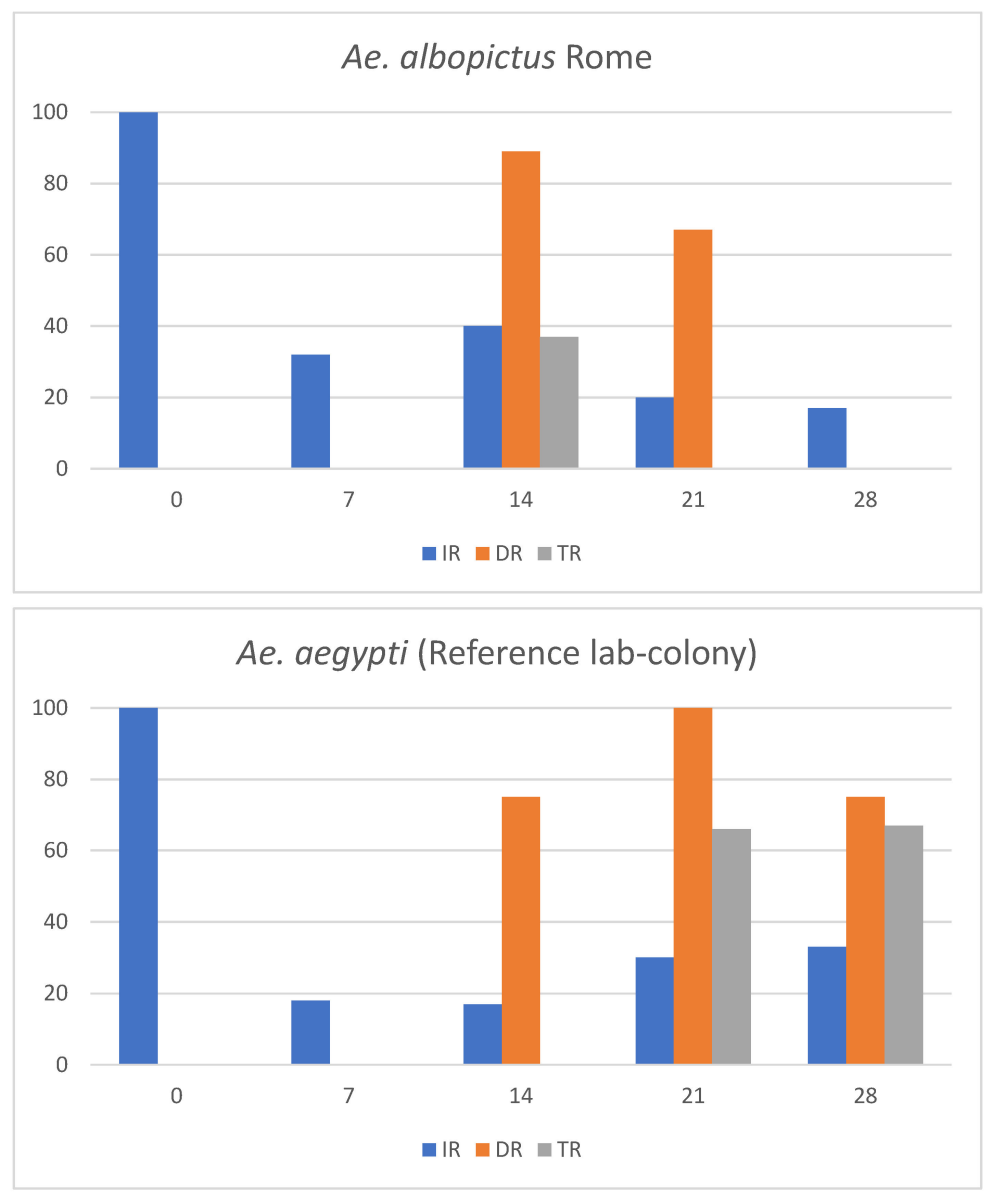
Infection (IR), dissemination (DR) and transmission rates (TR) in *Aedes albopictus* and *Ae. aegypti* colonies at different collection times postinfection with DENV-1.

Both *Ae. albopictus* field populations collected in Montecchio Maggiore and Rome were DENV free as all tested pools were negative. All tested *Aedes* populations showed susceptibility to DENV-1 infection, allowing the virus to replicate and spread to the salivary glands. The values of IR, DR, TR, and the mean viral titers are shown in Figure 1 and Table 1, respectively.

To confirm the ingestion of infectious viral particles, the engorged mosquitoes were analyzed immediately after the infectious blood meal. The results showed a viral titer of approximately 1 log_10_ PFU/mL in the tested specimens. The viral titers detected in the bodies of *Ae. albopictus* increased gradually in all mosquito specimens, reaching the highest mean values of 7.6 × 10^2^ PFU/mL 14 days post-infection (dpi) and 7.4 × 10^2^ PFU/mL 28 dpi in Rome and Montecchio Maggiore, respectively. In *Ae. aegypti* the highest mean titer was 1.4 × 10^3^ PFU/mL achieved 21 dpi (Table 1). Viral titers were higher in *Ae. aegypti* compared to *Ae. albopictus* populations, in particular at 21 dpi. The analysis indicated a progressive increase in IR over time for both *Ae. albopictus* populations, reaching the maximum value 14 dpi (40% Rome, 30% Montecchio Maggiore); subsequently, IR values decreased at 21 and 28 dpi. Conversely, *Ae. aegypti* showed a steady increase in IR throughout the observation period. DENV-1 was detected in legs plus wings from 14 dpi in all tested populations. All three mosquito groups showed high DR values (range 67–100%). DENV-1 was detected in saliva 14 dpi in both *Ae. albopictus* populations, highlighting a shorter extrinsic incubation period (EIP) compared to *Ae. aegypti*, in which DENV-1 was first detected in saliva 21 dpi. However, it was not possible to detect DENV-1 in the saliva after 21 dpi in *Ae. albopictus* from Rome and 28 dpi in *Ae. albopictus* from Montecchio Maggiore. In contrast, DENV-1 was detected in the saliva of *Ae. aegypti* until 28 dpi. Nevertheless, DENV-1 titers were relatively low in saliva (0.1 × 10^1^–0.5 × 10^1^ PFU/mL) in all analyzed populations (Table 1). Cumulative values of IR, DR and TR calculated from 7 to 28 dpi are reported in Table 2.

The *Aedes albopictus* population from Rome exhibited a higher IR (30%) compared to the other populations (18% for *Ae. albopictus* of Montecchio Maggiore and 22% for *Ae. aegypti*). However, the results highlighted a higher mean value of TR for *Ae. aegypti* (27%) compared to both *Ae. albopictus* populations (14% for Rome and 23% for Montecchio Maggiore). There was no evidence of vertical transmission in the offspring of the three *Aedes* populations.

## 4. Discussion

Dengue fever is a mosquito-borne tropical disease caused by DENV that has become a major public health problem in recent years causing approximately 390 million infections globally every year [10]. Several DENV strains have been implicated in autochthonous transmission in Europe since 2010. In recent years, DENV-1 and -2 have been the most prevalent serotypes in infections among European travelers [11]. Although *Ae. albopictus* is considered a secondary vector of DENV, its widespread distribution and high density in temperate areas represent a real risk for local outbreaks originating from imported cases. Until November 2023, 126 autochthonous/non-travel associated dengue cases have been reported in Europe in Italy (82), France (41), and Spain (3) [12,13]. In the summer of 2022, 65 autochthonous cases of DENV transmitted by *Ae. albopictus* were reported in France [14]. The unexpectedly high number of indigenous cases was however associated with nine distinct virus introduction and transmission events. This demonstrates the high risk of autochthonous transmission from imported cases but also the relatively small number of secondary cases associated with each individual index case. The first dengue outbreak in Italy occurred in August–September 2020. The outbreak was geographically limited to a small town in the Veneto region and caused very few indigenous human cases. *Aedes albopictus* was the mosquito vector incriminated as it is present and abundant in the area. This event could have been affected by various concomitant biotic and abiotic factors such as the lower vectorial competence of the *Ae. albopictus* population compared to the global primary vector, *Ae. Aegypti*, or the sudden change in atmospheric conditions with heavy rains and a sudden drop in temperatures which, together with vector control operations, led to a drastic decrease in the mosquito density. Another important determinant was the concomitant SARS-Cov-2 pandemic, during which the health authorities imposed a lockdown, effectively limiting people’s movements. This is confirmed by the fact that the people infected with DENV-1 during the outbreak were all relatives and neighbors [3]. It is known that the pathogenetic variation in virus strains, geographic distribution, vector abundance, and climatic factors influence vector susceptibility to DENV [15]. Studies on the role of possible mutations responsible for the better adaptation of arboviruses in *Ae. albopictus* have recently been conducted. Bellone et al. demonstrated that consecutive in vivo passages in *Ae. albopictus* resulted in the emergence of specific DENV-1 strains exhibiting increased infectivity for this vector both in vivo and in cultured mosquito cells. These alterations were facilitated by numerous adaptive mutations in the virus genome [2]. A comparative examination of the CHIKVs responsible for the Italian epidemics in 2007 and 2017 revealed that only the 2007 strain possessed the adaptive mutation E1 A226V for *Ae. albopictus*. These results highlight the significance of genomic studies in elucidating the potential role of the mutations in determining the adaptive capacity of a virus to different vectors [16]. Moreover, arboviruses adapt to secondary vectors as exemplified by the adaptation of Chikungunya virus to *Ae. albopictus* [17]. Therefore, a similar scenario cannot be ruled out for DENV. Our findings demonstrated that the Asiatic DENV-1 strain that was closely related to the 2020 Italian outbreak strain equally infected both *Ae. aegypti* and two local populations of *Ae. albopictus*. Interestingly, the virus reached the salivary glands of *Ae. albopictus* earlier than in *Ae. aegypti*. However, the virus survived longer in *Ae. aegypti* than in both populations of *Ae. albopictus*, suggesting a longer transmission potential of DENV-1 in *Ae. aegypti*. In our study, strains of *Ae. albopictus* collected in different areas of the country were infected with the same titer of DENV-1 in the same environment and experimental conditions to evaluate their vector competence. Of note, in agreement with our results, the DENV-1 strain used in the present study belonged to an established lineage during the DENV-1 outbreak in Singapore in 2013–14 and demonstrated efficient infection of an *Ae. Albopictus*-derived cell line [18]. The IR, DR, and TR rates in our study were not significantly different between *Ae. albopictus* and *Ae. aegypti* populations. In addition, the viral titers detected in the *Ae. albopictus* and *Ae. aegypti* populations were comparable. This result highlights the potentially important role of *Ae. albopictus* in the transmission of DENV-1 in non-endemic areas. In fact, the shorter EIP observed in *Ae. albopictus* compared to *Ae. aegypti* is noteworthy with regard to its potential to transmit DENV-1 effectively. Dengue outbreaks in France and Italy in 2023 confirm the increasing risk of DENV transmission in Europe potentially by *Ae. albopictus*, originating from imported cases. Data on the presence, abundance, seasonal fluctuations, and evaluation of vectorial competence of invasive mosquito species circulating in a non-endemic country are pivotal to assess the risk of arboviral transmission chains and, together with strengthened surveillance systems, for implementation of prevention and control measures to mitigate the adverse impacts on human health.

## Figures and Tables

**Table 1:** Infection rate (IR): number of positive bodies/number of tested fed females; dissemination rate (DR): number of positive legs plus wings/number of positive bodies; transmission rate (TR): number of positive saliva/number of positive bodies.

Species	Days Post Infection	IR	Main Value (PFU/mL)	DR	Main Value (PFU/mL)	TR	Main Value (PFU/mL)
*Ae. albopictus* Rome	0	11/11	1.6 × 10^1^	-		-	
	7	7/21	6.3 × 10^1^	0/7		0/7	
	14	9/22	7.6 × 10^2^	8/9	1.3 × 10^1^	3/9	0.4 × 10^1^
	21	3/15	3.0 × 10^2^	2/3	0.7 × 10^1^	0/3	
	28	2/12	4.1 × 10^2^	0/2		0/2	
*Ae. albopictus* Montecchio M.	0	12/12	1.9 × 10^1^	-		-	
	7	2/22	2.4 × 10^1^	0/2		0/2	
	14	7/23	3.8 × 10^2^	5/7	0.2 × 10^1^	2/7	0.1 × 10^1^
	21	3/15	6.4 × 10^2^	2/3	0.5 × 10^1^	1/3	0.5 × 10^1^
	28	1/12	7.4 × 10^2^	1/1	4.4 × 10^1^	0/1	
*Ae. aegypti* Reynosa	0	12/12	3.5 × 10^1^	-		-	
	7	4/22	1.9 × 10^1^	0/4		0/4	
	14	4/23	4.2 × 10^1^	3/4	0.5 × 10^1^	0/4	
	21	3/10	1.4 × 10^3^	3/3	1 × 10^1^	2/3	0.2 × 10^1^
	28	4/12	1.9 × 10^2^	3/4	3.3 × 10^1^	2/4	0.2 × 10^1^

**Table 2:** Infection rate (IR): number of positive bodies/number of tested fed females; dissemination rate (DR): number of positive legs plus wings/number of positive bodies; transmission rate (TR): number of positive saliva/number of positive bodies. Vector competence index (VCI): maximum value 1.0.

	*Ae. albopictus* Rome	*Ae. albopictus* Montecchio M.	*Ae. aegypti* Reference Lab-Colony
IR	30%	18%	22%
DR	48%	61%	60%
TR	14%	23%	27%
VCI	0.04	0.04	0.06

## Data Availability

The data presented in this study are available in the article.

## Supplementary Materials

The following supporting information can be downloaded at: https://www.mdpi.com/article/10.3390/v16020176/s1.
