# Supplementary material for: Assessing the Risk of Dengue Virus Local Transmission: Study on Vector Competence of Italian Aedes albopictus
Source: Viruses. 2024 Jan 24;16(2):176. doi: 10.3390/v16020176 (PMC10893310; doi:10.3390/v16020176)
Supplement: Supplementary file 1 [file viruses-16-00176-s001.zip › viruses-2824779-supplementary.pdf]

Alignment: C:\Users\claud\OneDrive\Desktop\seqdump.txt

```
                ....|....| ....|....| ....|....| ....|....| ....|....|
                        10         20         30         40         50
MZ291446.1      TTTCGAATCG GAAGCTTGCT TAACGTAGTT CTGACAGTTT TTTATTAGAG
MG097876.1      TTTCGAATCG GAAGCTTGCT TAACGTAGTT CTGACAGTTT TTTATTAGAG


                ....|....| ....|....| ....|....| ....|....| ....|....|
                        60         70         80         90        100
MZ291446.1      AGCAGATCTC TGATGAACAA CCAACGAAAA AAGACGGCTC GACCGTCTTT
MG097876.1      AGCAGATCTC TGATGAACAA CCAACGGAAA AAGACGGCTC GACCGTCTTT


                ....|....| ....|....| ....|....| ....|....| ....|....|
                       110        120        130        140        150
MZ291446.1      CAATATGCTG AAACGCGCGA GAAACCGCGT GTCAACTGTT TCACAGTTGG
MG097876.1      CAATATGCTG AAACGCGCGA GAAACCGCGT GTCAACTGTT TCACAGTTGG


                ....|....| ....|....| ....|....| ....|....| ....|....|
                       160        170        180        190        200
MZ291446.1      CGAAGAGATT CTCAAAAGGA TTGCTCTCAG GCCAAGGACC CATGAAACTG
MG097876.1      CGAAGAGATT CTCAAAAGGA TTGCTCTCAG GCCAAGGACC CATGAAACTG


                ....|....| ....|....| ....|....| ....|....| ....|....|
                       210        220        230        240        250
MZ291446.1      GTGATGGCTT TCATAGCATT CCTAAGATTT CTAGCCATAC CCCCAACAGC
MG097876.1      GTGATGGCTT TCATAGCATT CCTAAGATTT CTAGCCATAC CCCCAACAGC


                ....|....| ....|....| ....|....| ....|....| ....|....|
                       260        270        280        290        300
MZ291446.1      AGGAATTTTG GCTAGATGGG GCTCATTCAA GAAGAATGGA GCGATCAAAG
MG097876.1      AGGAATTTTG GCTAGATGGG GTTCATTCAA GAAGAATGGA GCGATCAAAG


                ....|....| ....|....| ....|....| ....|....| ....|....|
                       310        320        330        340        350
MZ291446.1      TGCTACGGGG TTTCAAGAAA GAAATCTCAA GCATGTTGAA CATAATGAAT
MG097876.1      TGCTACGGGG TTTCAAGAAA GAAATCTCAA GCATGTTGAA TATAATGAAT


                ....|....| ....|....| ....|....| ....|....| ....|....|
                       360        370        380        390        400
MZ291446.1      AGAAGGAAAA GATCTGTGAC CATGCTCCTT ATGCTGATGC CTACAGCCTT
MG097876.1      AGAAGGAAAA GATCTGTGAC CATGCTCCTT ATGCTGATGC CTACAGCCTT


                ....|....| ....|....| ....|....| ....|....| ....|....|
                       410        420        430        440        450
MZ291446.1      GGCATTCCAT TTGACTACAC GAGGGGGAGA GCCGCACATG ATAGTCAGCA
MG097876.1      GGCGTTCCAC TTGACTACAC GAGGGGGAGA GCCGCACATG ATAGTCAGCA


                ....|....| ....|....| ....|....| ....|....| ....|....|
                       460        470        480        490        500
MZ291446.1      AGCAGGAAAG AGGAAAGTCA CTTTTGTTTA AGACCTCAGC AGGTGTCAAT
MG097876.1      AGCAGGAAAG AGGAAAGTCT CTTTTGTTTA AGACCTCAGC AGGTGTCAAC


                ....|....| ....|....| ....|....| ....|....| ....|....|
                       510        520        530        540        550
MZ291446.1      ATGTGCACCC TCATAGCGAT GGATTTGGGA GAGTTATGTG AGGACACAAT
MG097876.1      ATGTGCACCC TTATAGCGAT GGATTTGGGA GAGTTATGTG AGGACACAAT


                ....|....| ....|....| ....|....| ....|....| ....|....|
                       560        570        580        590        600
MZ291446.1      GACTTACAAA TGCCCTCGAA TCACTGAGGC GGAACCAGAT GACGTTGATT
MG097876.1      GACTTACAAA TGCCCTCGAA TCACTGAGGC GGAACCAGAT GACGTTGATT
```

```
                ....|....| ....|....| ....|....| ....|....| ....|....|
                       610        620        630        640        650
MZ291446.1      GTTGGTGCAA TGCCACAGAC ACATGGGTGA CCTATGGAAC ATGTTCCCAA
MG097876.1      GTTGGTGCAA TGCCACAGAC ACATGGGTGA CCTATGGAAC ATGTTCCCAA


                ....|....| ....|....| ....|....| ....|....| ....|....|
                       660        670        680        690        700
MZ291446.1      ACTGGCGAGC ACCGACGAGA CAAACGTTCC GTTGCACTGG CACCACACGT
MG097876.1      ACTGGCGAGC ACCGACGAGA CAAACGTTCC GTCGCACTGG CCCCACACGT


                ....|....| ....|....| ....|....| ....|....| ....|....|
                       710        720        730        740        750
MZ291446.1      GGGACTTGGT TTGGAAACAA GAACTGAAAC GTGGATGTCC TCTGAAGGCG
MG097876.1      GGGACTTGGT TTGGAAACAA GAACCGAAAC GTGGATGTCC TCCGAAGGAG


                ....|....| ....|....| ....|....| ....|....| ....|....|
                       760        770        780        790        800
MZ291446.1      CTTGGAAACA GATACAAAGA GTGGAGACTT GGGCCCTGAG ACACCCAGGA
MG097876.1      CTTGGAAACA GATACAAAGA GTGGAGACTT GGGCCCTGAG ACACCCAGGA


                ....|....| ....|....| ....|....| ....|....| ....|....|
                       810        820        830        840        850
MZ291446.1      TTCACGGTGA TAGCCCTTTT TCTAGCACAT GCCATAGGAA CATCCATCAC
MG097876.1      TTCACGGCGA TAGCCCTTTT TTTAGCACAT GCCATAGGAA CATCCATCAC


                ....|....| ....|....| ....|....| ....|....| ....|....|
                       860        870        880        890        900
MZ291446.1      CCAGAAAGGG ATTATCTTCA TTTTGTTAAT GCTGGTTACA CCATCTATGG
MG097876.1      CCAGAAAGGG ATTATCTTCA TTTTGTTAAT GCTGGTTACA CCATCCATGG


                ....|....| ....|....| ....|....| ....|....| ....|....|
                       910        920        930        940        950
MZ291446.1      CCATGCGATG CGTGGGAATA GGCAGCAGGG ACTTCGTGGA AGGACTGTCA
MG097876.1      CCATGCGATG CGTGGGAATA GGCAGCAGGG ACTTCGTGGA AGGACTGTCA


                ....|....| ....|....| ....|....| ....|....| ....|....|
                       960        970        980        990       1000
MZ291446.1      GGAGCAACTT GGGTAGATGT GGTGTTGGAA CATGGAAGTT GCGTCACTAC
MG097876.1      GGAGCAACTT GGGTAGATGT GGTGTTGGAA CATGGAAGTT GCGTTACTAC


                ....|....| ....|....| ....|....| ....|....| ....|....|
                      1010       1020       1030       1040       1050
MZ291446.1      CATGGCAAAA GACAAACCAA CATTGGACAT TGAACTCTTG AAGACGGAAG
MG097876.1      CATGGCAAAA GATAAACCAA CATTGGACAT TGAACTCTTG AAGACGGAAG


                ....|....| ....|....| ....|....| ....|....| ....|....|
                      1060       1070       1080       1090       1100
MZ291446.1      TCACAAACCC TGCCGTCCTG CGCAAACTGT GCATTGAAGC TAAAATATCA
MG097876.1      TCACAAACCC TGCCGTCTTG CGCAAACTGT GCATTGAAGC TAAAATATCA


                ....|....| ....|....| ....|....| ....|....| ....|....|
                      1110       1120       1130       1140       1150
MZ291446.1      AACACCACCA CTGACTCAAG ATGTCCAACA CAAGGAGAAG CCACACTGGT
MG097876.1      AACACCACCA CCGACTCAAG ATGTCCAACA CAAGGAGAAG CCACATTGGT


                ....|....| ....|....| ....|....| ....|....| ....|....|
                      1160       1170       1180       1190       1200
MZ291446.1      GGAAGAACAA GACGCGAACT TTGTGTGTCG ACGAACGTTT GTGGACAGAG
MG097876.1      GGAAGAACAA GACGCGAACT TTGTGTGTCG ACGAACGTTT GTGGACAGAG
```

```
               ....|....| ....|....| ....|....| ....|....| ....|....|
                     1210       1220       1230       1240       1250
MZ291446.1     GCTGGGGCAA TGGATGTGGG CTCTTCGGAA AAGGAAGCCT TATAACGTGT
MG097876.1     GCTGGGGCAA TGGCTGTGGG CTTTTCGGAA AAGGAAGCCT TATAACGTGT

               ....|....| ....|....| ....|....| ....|....| ....|....|
                     1260       1270       1280       1290       1300
MZ291446.1     GCCAAGTTCA AGTGTGTGAC AAAACTGGAA GGAAAAATAG TTCAATATGA
MG097876.1     GCTAAGTTCA AGTGTGTGAC AAAACTGGAA GGAAAAATAG TTCAATATGA

               ....|....| ....|....| ....|....| ....|....| ....|....|
                     1310       1320       1330       1340       1350
MZ291446.1     AAACTTGAAA TATTCAGTAA TAGTCACCGT CCACACTGGA GACCAGCACC
MG097876.1     AAACTTGAAA TATTCAGTAA TAGTCACCGT CCACACTGGA GACCAGCACC

               ....|....| ....|....| ....|....| ....|....| ....|....|
                     1360       1370       1380       1390       1400
MZ291446.1     AGGTGGGAAA TGAAAGCACA GAACATGGGA CAACTGCAAC CATAACACCT
MG097876.1     AGGTGGGAAA TGAAAGCACA GAACATGGGA CAACTGCAAC CATAACACCT

               ....|....| ....|....| ....|....| ....|....| ....|....|
                     1410       1420       1430       1440       1450
MZ291446.1     CAAGCTCCTA CGACAGAAAT ACAGCTGACC GACTACGGAG CTCTCACATT
MG097876.1     CAAGCTCCTA CGACAGAAAT ACAGCTGACC GACTACGGAG CTCTTACATT

               ....|....| ....|....| ....|....| ....|....| ....|....|
                     1460       1470       1480       1490       1500
MZ291446.1     GGATTGTTCA CCTAGAACAG GACTGGACTT CAATGAAATG GTGTTGTTGA
MG097876.1     GGATTGTTCA CCTAGAACAG GACTGGACTT CAATGAAATG GTGTTGTTGA

               ....|....| ....|....| ....|....| ....|....| ....|....|
                     1510       1520       1530       1540       1550
MZ291446.1     CAATGAAAGA AAAGTCATGG CTAGTCCACA AACAATGGTT CCTAGACCTA
MG097876.1     CAATGAAAGA AAAGTCATGG CTAGTCCACA AACAATGGTT TCTAGACCTA

               ....|....| ....|....| ....|....| ....|....| ....|....|
                     1560       1570       1580       1590       1600
MZ291446.1     CCACTGCCTT GGACCTCGGG AGCTTCAACA TTACAAGAAA CTTGGAACAG
MG097876.1     CCACTGCCTT GGACCTCGGG AGCTTCAACA TCACAAGAAA CTTGGAACAG

               ....|....| ....|....| ....|....| ....|....| ....|....|
                     1610       1620       1630       1640       1650
MZ291446.1     ACAAGATTTG CTGGTGACAT TTAAGACAGC TCATGCAAAG AAGCAGGAAG
MG097876.1     ACAAGATTTG CTGGTGACAT TTAAGACAGC TCATGCAAAG AAGCAGGAAG

               ....|....| ....|....| ....|....| ....|....| ....|....|
                     1660       1670       1680       1690       1700
MZ291446.1     TAGTCGTACT AGGATCACAA GAAGGAGCAA TGCACACTGC GTTGACCGGA
MG097876.1     TAGTCGTACT AGGATCACAA GAAGGAGCAA TGCACACTGC GTTGACCGGA

               ....|....| ....|....| ....|....| ....|....| ....|....|
                     1710       1720       1730       1740       1750
MZ291446.1     GCGACGGAAA TCCAAACGTC TGGAACGACA ACAATTTTTG CAGGACACTT
MG097876.1     GCGACGGAAA TCCAAACGTC TGGAACGACA ACAATTTTTG CAGGACACTT

               ....|....| ....|....| ....|....| ....|....| ....|....|
                     1760       1770       1780       1790       1800
MZ291446.1     GAAATGTAGA CTAAAGATGG ACAAACTGAC TCTAAAAGGG ATGTCATATG
MG097876.1     GAAATGTAGA TTAAAGATGG ACAAACTGAC TCTAAAAGGG ATGTCATATG
```

```
               ....|....| ....|....| ....|....| ....|....| ....|....|
                      1810       1820       1830       1840       1850
MZ291446.1     TGATGTGCAC AGGCTCATTC AAGCTAGAGA AAGAAGTGGC TGAGACCCAG
MG097876.1     TGATGTGCAC AGGCTCATTC AAGCTAGAGA AAGAAGTGGC TGAGACCCAG

               ....|....| ....|....| ....|....| ....|....| ....|....|
                      1860       1870       1880       1890       1900
MZ291446.1     CATGGAACCG TTCTAGTGCA GATTAAATAC GAAGGAGCAG ATGCACCATG
MG097876.1     CATGGAACCG TTCTAGTGCA GATTAAATAC GAAGGAACAG ATGCACCATG

               ....|....| ....|....| ....|....| ....|....| ....|....|
                      1910       1920       1930       1940       1950
MZ291446.1     CAAGATCCCT TTTTCGACCC AAGATGAAAA AGGAGTAACC CAGAATGGGA
MG097876.1     CAAGATCCCT TTTTTGACCC AAGATGAAAA AGGAGTAACC CAGAATGGGA

               ....|....| ....|....| ....|....| ....|....| ....|....|
                      1960       1970       1980       1990       2000
MZ291446.1     GATTGATAAC AGCCAACCCC ATAGTCACTG ACAAAGAAAA ACCAGTCAAC
MG097876.1     GATTGATAAC AGCCAACCCC ATAGTCTCTG ACAAAGAAAA ACCAGTCAAC

               ....|....| ....|....| ....|....| ....|....| ....|....|
                      2010       2020       2030       2040       2050
MZ291446.1     ATTGAGGCAG AACCGCCTTT TGGTGAGAGT TATATCGTGA TAGGAGCAGG
MG097876.1     ATTGAGGCAG AACCGCCTTT TGGTGAGAGT TACATCGTGA TAGGAGCAGG

               ....|....| ....|....| ....|....| ....|....| ....|....|
                      2060       2070       2080       2090       2100
MZ291446.1     TGAAAAGGCT TTGAAACTAA GCTGGTTCAA GAAAGGAAGC AGCATAGGGA
MG097876.1     TGAAAAAGCT TTGAAACTAA GCTGGTTCAA GAAAGGAAGC AGCATAGGGA

               ....|....| ....|....| ....|....| ....|....| ....|....|
                      2110       2120       2130       2140       2150
MZ291446.1     AAATGTTTGA GGCAACTGCC AGAGGAGCAC GAAGGATGGC CATACTGGGA
MG097876.1     AAATGTTTGA GGCAACTGCC AGAGGAGCAC GAAGGATGGC CATACTGGGA

               ....|....| ....|....| ....|....| ....|....| ....|....|
                      2160       2170       2180       2190       2200
MZ291446.1     GACACCGCAT GGGACTTTGG TTCTATAGGA GGAGTGTTCA CGTCTGTTGG
MG097876.1     GACACCGCAT GGGACTTTGG TTCTATAGGA GGAGTGTTCA CGTCTGTTGG

               ....|....| ....|....| ....|....| ....|....| ....|....|
                      2210       2220       2230       2240       2250
MZ291446.1     AAAACTAGTA CACCAGATTT TTGGAACTGC ATATGGAGTT TTGTTCAGCG
MG097876.1     AAAATTAGTA CACCAGATTT TTGGAACTGC ATATGGAGTT TTGTTCAGCG

               ....|....| ....|....| ....|....| ....|....| ....|....|
                      2260       2270       2280       2290       2300
MZ291446.1     GTGTTTCCTG GACCATGAAA ATAGGAATAG GGGTTCTGCT GACATGGCTA
MG097876.1     GTGTTTCCTG GACCATGAAA ATAGGAATAG GGGTTCTGCT GACATGGCTA

               ....|....| ....|....| ....|....| ....|....| ....|....|
                      2310       2320       2330       2340       2350
MZ291446.1     GGATTAAACT CAAGGAGCAC GTCCCTTTCG ATGACGTGCA TTGCAGTCGG
MG097876.1     GGATTAAACT CAAGGAGCAC GTCCCTTTCG ATGACGTGCA TTGCAGTTGG

               ....|....| ....|....| ....|....| ....|....| ....|....|
                      2360       2370       2380       2390       2400
MZ291446.1     CCTAGTAACG CTATACTTAG GAGTCATGGT TCAGGCGGAT TCAGGATGTG
MG097876.1     CCTAGTAACG CTATACTTAG GAGTCATGGT TCAGGCGGAT TCAGGATGTG

               ....|....| ....|....| ....|....| ....|....| ....|....|
```

```
                2410       2420       2430       2440       2450
MZ291446.1   TAATCAATTG GAAAGGTAGA GAACTCAAAT GTGGGAGTGG CATTTTTGTC
MG097876.1   TAATTAATTG GAAAGGTAGA GAACTCAAAT GTGGAAGTGG CATTTTTGTC


             ....|....| ....|....| ....|....| ....|....| ....|....|
                2460       2470       2480       2490       2500
MZ291446.1   ACTAATGAAG TTCACACTTG GACAGAGCAA TACAAATTTC AAGCTGATTC
MG097876.1   ACCAATGAAG TTCACACTTG GACAGAGCAA TACAAATTTC AAGCTGATTC


             ....|....| ....|....| ....|....| ....|....| ....|....|
                2510       2520       2530       2540       2550
MZ291446.1   CCCAAAGAGA CTATCAGCAG CCATTGGGAA GGCATGGGAG GAGGGTGTGT
MG097876.1   CCCAAAGAGA TTATCAGCAG CCATTGGGAA GGCATGGGAG GAGGGTGTGT


             ....|....| ....|....| ....|....| ....|....| ....|....|
                2560       2570       2580       2590       2600
MZ291446.1   GTGGAATTCG ATCAGCAACC CGTCTCGAGA ACATCATGTG GAAGCAAATA
MG097876.1   GTGGAATTCG ATCAGCAACT CGTCTCGAGA ACATCATGTG GAAGCAAATA


             ....|....| ....|....| ....|....| ....|....| ....|....|
                2610       2620       2630       2640       2650
MZ291446.1   TCAAATGAAT TGAACCACAT CTTGCTTGAA AATGACATGA AATTCACAGT
MG097876.1   TCAAATGAAT TGAACCACAT CTTGCTTGAA AATGACATGA AATTTACAGT


             ....|....| ....|....| ....|....| ....|....| ....|....|
                2660       2670       2680       2690       2700
MZ291446.1   GGTTGTAGGA GATGTTGCTG GGATCTTGGC TCAAGGGAAA AAAATGATTA
MG097876.1   GGTTGTAGGA GATGTTGCTG GGATCTTGGC TCAAGGAAAA AAGATGATTA


             ....|....| ....|....| ....|....| ....|....| ....|....|
                2710       2720       2730       2740       2750
MZ291446.1   GGCCACAACC CATGGAACAC AAATACTCGT GGAAAAGCTG GGGAAAAGCT
MG097876.1   GGCCACAACC CATGGAACAC AAATACTCGT GGAAAAGCTG GGGAAAGGCT


             ....|....| ....|....| ....|....| ....|....| ....|....|
                2760       2770       2780       2790       2800
MZ291446.1   AAAATCATAG GGGCAGATGT ACAGAACACC ACCTTTATCA TCGACGGCCC
MG097876.1   AAAATCATAG GGGCAGATGT ACAGAACACC ACCTTTATCA TCGACGGCCC


             ....|....| ....|....| ....|....| ....|....| ....|....|
                2810       2820       2830       2840       2850
MZ291446.1   AAACACCCCA GAATGCCCTG ATGATCAAAG AGCATGGAAC ATTTGGGAAG
MG097876.1   AAACACCCCA GAATGCCCTG ATGATCAAAG AGCATGGAAC ATTTGGGAAG


             ....|....| ....|....| ....|....| ....|....| ....|....|
                2860       2870       2880       2890       2900
MZ291446.1   TTGAGGATTA TGGATTTGGA ATTTTCACGA CAAACATATG GCTGAAATTG
MG097876.1   TTGAAGATTA TGGATTTGGA ATTTTCACGA CAAACATATG GCTGAAATTG


             ....|....| ....|....| ....|....| ....|....| ....|....|
                2910       2920       2930       2940       2950
MZ291446.1   CGTGACTCCT ACACCCAAGT GTGTGACCAC CGGCTAATGT CAGCTGCCAT
MG097876.1   CGCGACTCCT ACACCCAAGT GTGTGACCAC CGGCTAATGT CAGCTGCCAT


             ....|....| ....|....| ....|....| ....|....| ....|....|
                2960       2970       2980       2990       3000
MZ291446.1   CAAAGACAGC AAGGCAGTTC ATGCTGACAT GGGGTACTGG ATAGAAAGTG
MG097876.1   CAAGGACAGC AAGGCAGTTC ACGCTGACAT GGGGTACTGG ATAGAAAGTG


             ....|....| ....|....| ....|....| ....|....| ....|....|
                3010       3020       3030       3040       3050
```

```
MZ291446.1   AAAAGAATGA GACCTGGAAG CTGGCAAGAG CCTCTTTCAT AGAAGTCAAA
MG097876.1   AAAAGAACGA GACCTGGAAG CTGGCAAGAG CCTCTTTCAT AGAAGTCAAA


             ....|....| ....|....| ....|....| ....|....| ....|....|
                   3060       3070       3080       3090       3100
MZ291446.1   ACGTGTATCT GGCCAAAATC TCACACTCTA TGGAGCAATG GAGTTCTGGA
MG097876.1   ACATGTATCT GGCCAAAATC TCACACTCTA TGGAGCAATG GAGTTCTGGA


             ....|....| ....|....| ....|....| ....|....| ....|....|
                   3110       3120       3130       3140       3150
MZ291446.1   AAGTGAAATG ATAATTCCAA AGATCTATGG AGGACCAATA TCTCAGCACA
MG097876.1   AAGTGAAATG ATAATTCCAA AGATCTATGG AGGACCAATA TCTCAGCACA


             ....|....| ....|....| ....|....| ....|....| ....|....|
                   3160       3170       3180       3190       3200
MZ291446.1   ACTACAGACC AGGATATTTC ACACAAACGG CAGGGCCGTG GCATCTAGGC
MG097876.1   ACTACAGACC AGGATATTTC ACACAAACGG CAGGGCCGTG GCACCTAGGC


             ....|....| ....|....| ....|....| ....|....| ....|....|
                   3210       3220       3230       3240       3250
MZ291446.1   AAGTTGGAAC TGGATTTTGA TTTGTGTGAG GGAACCACGG TTGTTGTGGA
MG097876.1   AAGTTGGAAC TGGATTTTGA TTTGTGTGAG GGTACCACGG TTGTTGTGGA


             ....|....| ....|....| ....|....| ....|....| ....|....|
                   3260       3270       3280       3290       3300
MZ291446.1   TGAACATTGT GGAAATCGAG GACCATCTCT TAGGACCACA ACAGTCACAG
MG097876.1   TGAACATTGT GGAAATCGAG GACCATCTCT TAGGACCACA ACAGTCACAG


             ....|....| ....|....| ....|....| ....|....| ....|....|
                   3310       3320       3330       3340       3350
MZ291446.1   GAAAGATAAT TCATGAATGG TGTTGCAGAT CTTGTACGCT ACCACCCTTA
MG097876.1   GAAAGATAAT TCATGAATGG TGTTGCAGAT CTTGTACGCT ACCACCCTTA


             ....|....| ....|....| ....|....| ....|....| ....|....|
                   3360       3370       3380       3390       3400
MZ291446.1   CGTTTCAGAG GAGAAGATGG GTGCTGGTAC GGTATGGAAA TCAGACCAGT
MG097876.1   CGTTTCAGAG GAGAAGATGG GTGCTGGTAC GGTATGGAAA TCAGACCAGT


             ....|....| ....|....| ....|....| ....|....| ....|....|
                   3410       3420       3430       3440       3450
MZ291446.1   CAAGGAAAAG GAAGAGAATC TAGTCAAATC AATGGTCTCT GCAGGGTCAG
MG097876.1   CAAGGAAAAG GAAGAGAATC TAGTCAAATC AATGGTCTCT GCAGGGTCAG


             ....|....| ....|....| ....|....| ....|....| ....|....|
                   3460       3470       3480       3490       3500
MZ291446.1   GGGAAGTGGA CAGCTTTTCA CTAGGACTGT TATGCATATC AATAATGATC
MG097876.1   GGGAAGTGGA CAGCTTTTCA CTAGGACTGT TATGCATATC AATAATGATC


             ....|....| ....|....| ....|....| ....|....| ....|....|
                   3510       3520       3530       3540       3550
MZ291446.1   GAAGAGGTGA TGAGATCCAG ATGGAGTAGA AAAATGCTGA TGACTGGAAT
MG097876.1   GAAGAGGTGA TGAGATCCAG ATGGAGTAGA AAAATGCTGA TGACTGGAAC


             ....|....| ....|....| ....|....| ....|....| ....|....|
                   3560       3570       3580       3590       3600
MZ291446.1   ACTGGCTGTG TTCTTCCTTC TCATAATGGG ACAATTGACA TGGAATGATT
MG097876.1   ACTGGCTGTG TTCTTCCTTC TCATAATGGG ACAACTGACA TGGAATGATC


             ....|....| ....|....| ....|....| ....|....| ....|....|
                   3610       3620       3630       3640       3650
MZ291446.1   TGATCAGATT ATGCATCATG GTTGGAGCCA ACGCTTCCGA CAGGATGGGG
```

```
MG097876.1   TGATTAGATT ATGCATCATG GTTGGAGCCA ACGCTTCCGA CAGGATGGGG

             ....|....| ....|....| ....|....| ....|....| ....|....|
                   3660       3670       3680       3690       3700
MZ291446.1   ATGGGAACGA CCTACCTAGC TCTGATGGCT ACTTTTAAAA TGAGACCAAT
MG097876.1   ATGGGAACGA CGTACCTAGC TCTGATGGCC ACTTTTAAAA TGAGACCAAT

             ....|....| ....|....| ....|....| ....|....| ....|....|
                   3710       3720       3730       3740       3750
MZ291446.1   GTTTGCCGTA GGGCTACTAT TCCGCAGACT AACATCCAGA GAAGTTCTTC
MG097876.1   GTTTGCCGTA GGGCTACTAT TCCGCAGACT AACATCCAGA GAAGTTCTTC

             ....|....| ....|....| ....|....| ....|....| ....|....|
                   3760       3770       3780       3790       3800
MZ291446.1   TTCTAACAAT TGGATTGAGT CTAGTGGCAT CTGTGGAGTT ACCAAATTCC
MG097876.1   TTCTAACAAT TGGATTGAGT CTAGTGGCAT CTGTGGAGTT ACCAAATTCC

             ....|....| ....|....| ....|....| ....|....| ....|....|
                   3810       3820       3830       3840       3850
MZ291446.1   TTGGAGGAGC TGGGGGATGG ACTTGCAATG GGCATTATGA TCTTAAAATT
MG097876.1   TTGGAGGAGC TGGGGGATGG ACTTGCAATG GGCATTATGA TCTTAAAATT

             ....|....| ....|....| ....|....| ....|....| ....|....|
                   3860       3870       3880       3890       3900
MZ291446.1   ATTGACTGAC TTTCAATCAC ATCAGCTGTG GGCTACCTTG CTGTCCTTGA
MG097876.1   ATTGACTGAC TTTCAATCAC ATCAGCTGTG GGCTACCTTG CTGTCCTTGA

             ....|....| ....|....| ....|....| ....|....| ....|....|
                   3910       3920       3930       3940       3950
MZ291446.1   CATTTATCAA AACAACGTTT TCCTTGCACT GTGCATGGAA GACTATGGCT
MG097876.1   CATTTATCAA AACAACGTTT TCCTTGCACT ATGCATGGAA GACAATGGCT

             ....|....| ....|....| ....|....| ....|....| ....|....|
                   3960       3970       3980       3990       4000
MZ291446.1   ATGGTACTGT CAATTGTATC TCTCTTCCCC TTATGCCTGT CCACGACCTC
MG097876.1   ATGGTATTGT CAATTGTATC TCTCTTCCCC TTATGCCTGT CCACGACCTC

             ....|....| ....|....| ....|....| ....|....| ....|....|
                   4010       4020       4030       4040       4050
MZ291446.1   CCAAAAAACA ACATGGCTTC CGGTGCTGTT GGGATCTCTT GGATGCAAAC
MG097876.1   CCAAAAAACA ACATGGCTTC CAGTGCTGTT GGGATCTCTT GGATGCAAAC

             ....|....| ....|....| ....|....| ....|....| ....|....|
                   4060       4070       4080       4090       4100
MZ291446.1   CACTAACCAT GTTTCTTATA GCAGAAAACA AAATCTGGGG AAGGAAAAGT
MG097876.1   CACTAACCAT GTTTCTTATA GCAGAAAACA AAATTTGGGG AAGGAAAAGT

             ....|....| ....|....| ....|....| ....|....| ....|....|
                   4110       4120       4130       4140       4150
MZ291446.1   TGGCCCCTCA ATGAAGGAAT CATGGCTGTT GGAATAGTCA GTATCCTACT
MG097876.1   TGGCCCCTCA ATGAAGGAAT CATGGCTGTT GGAATAGTCA GCATCCTACT

             ....|....| ....|....| ....|....| ....|....| ....|....|
                   4160       4170       4180       4190       4200
MZ291446.1   AAGTTCACTC CTCAAAAATG ATGTGCCGCT AGCTGGGCCA TTGATAGCTG
MG097876.1   AAGTTCATTC CTCAAAAATG ATGTGCCGCT AGCTGGGCCA TTGATAGCTG

             ....|....| ....|....| ....|....| ....|....| ....|....|
                   4210       4220       4230       4240       4250
MZ291446.1   GAGGCATGCT AATAGCATGT TATGTCATAT CTGGAAGCTC AGCCGATCTA
MG097876.1   GAGGCATGCT AATAGCATGT TATGTCATAT CTGGAAGCTC AGCCGATCTA
```

```
                ....|....| ....|....| ....|....| ....|....| ....|....|
                        4260       4270       4280       4290       4300
MZ291446.1      TCACTAGAGA AAGCGGCTGA GGTCTCCTGG GAAAAAGAAG CAGAACACTC
MG097876.1      TCACTAGAGA AAGCGGCTGA GGTCTCCTGG GAAGAAGAAG CAGAACACTC

                ....|....| ....|....| ....|....| ....|....| ....|....|
                        4310       4320       4330       4340       4350
MZ291446.1      TGGTGCCTCA CACAACATAT TAGTGGAGGT CCAAGATGAT GGAACCATGA
MG097876.1      TGGTGCCTCA CACAACATAT TAGTGGAGGT CCAAGATGAT GGAACCATGA

                ....|....| ....|....| ....|....| ....|....| ....|....|
                        4360       4370       4380       4390       4400
MZ291446.1      AGATAAAGGA TGAAGAGAGA GATGACACGC TAACCATTCT CCTTAAAGCA
MG097876.1      AGATAAAGGA TGAAGAGAGA GATGACACGC TAACCATTCT CCTTAAAGCA

                ....|....| ....|....| ....|....| ....|....| ....|....|
                        4410       4420       4430       4440       4450
MZ291446.1      ACTCTGCTAG CAATTTCAGG GGTGTACCCA TTGTCAATAC CAGCGACCCT
MG097876.1      ACTCTGCTAG CAGTTTCAGG GGTGTACCCA TTATCAATAC CAGCGACCCT

                ....|....| ....|....| ....|....| ....|....| ....|....|
                        4460       4470       4480       4490       4500
MZ291446.1      TTTTGTGTGG TACTTTTGGC AGAAAAAGAA ACAGAGATCT GGAGTGTTAT
MG097876.1      TTTTGTGTGG TACTTTTGGC AGAAAAAGAA ACAGAGATCT GGAGTGTTAT

                ....|....| ....|....| ....|....| ....|....| ....|....|
                        4510       4520       4530       4540       4550
MZ291446.1      GGGACACACC CAGCCCTCCA GAAGTGGAAA GAGCAGTTCT TGATGATGGC
MG097876.1      GGGACACACC TAGCCCTCCA GAAGTGGAAA GAGCAGTTCT TGATGATGGC

                ....|....| ....|....| ....|....| ....|....| ....|....|
                        4560       4570       4580       4590       4600
MZ291446.1      ATCTATAGAA TCATGCAGAG AGGACTGTTG GGCAGGTCTC AAGTGGGGGT
MG097876.1      ATCTATAGAA TCATGCAGAG AGGACTGTTG GGCAGGTCCC AAGTGGGGGT

                ....|....| ....|....| ....|....| ....|....| ....|....|
                        4610       4620       4630       4640       4650
MZ291446.1      AGGAGTGTTT CAAGGAAACG TGTTCCACAC AATGTGGCAT GTCACCAGGG
MG097876.1      AGGAGTGTTT CAAGAAAACG TGTTCCACAC AATGTGGCAT GTCACCAGGG

                ....|....| ....|....| ....|....| ....|....| ....|....|
                        4660       4670       4680       4690       4700
MZ291446.1      GAGCTGTACT CATGTACCAA GGGAAGAGAC TGGAACCGAG CTGGGCCAGT
MG097876.1      GAGCTGTACT CATGTATCAA GGGAAGAGAC TGGAACCGAG CTGGGCCAGT

                ....|....| ....|....| ....|....| ....|....| ....|....|
                        4710       4720       4730       4740       4750
MZ291446.1      GTCAAAAAAG ACCTGATCTC ATATGGAGGA GGTTGGAGGC TTCAAGGATC
MG097876.1      GTCAAAAAAG ACCTAATCTC ATATGGAGGA GGTTGGAGGC TTCAAGGATC

                ....|....| ....|....| ....|....| ....|....| ....|....|
                        4760       4770       4780       4790       4800
MZ291446.1      CTGGAACACG GGAGAAGAAG TGCAGGTGAT TGCTGTTGAA CCAGGGAAAA
MG097876.1      CTGGAACACG GGAGAAGAAG TGCAGGTGAT TGCTGTTGAA CCAGGGAAAA

                ....|....| ....|....| ....|....| ....|....| ....|....|
                        4810       4820       4830       4840       4850
MZ291446.1      ACCCCAAAAA TGTACAAACA GCGCCGGGCA CTTTTAAGAC CCCTGAAGGC
MG097876.1      ACCCCAAAAA TGTACAAACA GCGCCGGGCA CCTTTAAGAC CCCTGAAGGC
```

```
                ....|....| ....|....| ....|....| ....|....| ....|....|
                        4860       4870       4880       4890       4900
MZ291446.1   GAAGTTGGAG CTATTGCCCT AGATTTTAAA CCCGGCACAT CTGGATCTCC
MG097876.1   GAAGTTGGAG CCATTGCCCT AGATTTTAAA CCCGGCACAT CTGGATCTCC

                ....|....| ....|....| ....|....| ....|....| ....|....|
                        4910       4920       4930       4940       4950
MZ291446.1   CATCGTGAAC AGAGAAGGGA AAATAGTAGG TCTTTATGGA AATGGAGTGG
MG097876.1   CATCGTGAAC AGAGAAGGGA AAATAGTAGG TCTTTATGGA AATGGAGTAG

                ....|....| ....|....| ....|....| ....|....| ....|....|
                        4960       4970       4980       4990       5000
MZ291446.1   TGACAACAAG TGGAACCTAC GTCAGTGCCA TAGCTCAAGC TAAAGCATCA
MG097876.1   TGACAACAAG TGGAACCTAC GTCAGTGCCA TAGCTCAAGC CAAAGCATCA

                ....|....| ....|....| ....|....| ....|....| ....|....|
                        5010       5020       5030       5040       5050
MZ291446.1   CAAGAGGGGC CCCTACCAGA GATTGAAGAT GAGGTGTTTA GGAAAAGAAA
MG097876.1   CAAGAAGGGC CCCTACCAGA GATTGAAGAC GAGGTGTTTA AGAAAAGAAA

                ....|....| ....|....| ....|....| ....|....| ....|....|
                        5060       5070       5080       5090       5100
MZ291446.1   CTTAACAATA ATGGATCTGC ATCCAGGATC GGGAAAAACA AGAAGATACC
MG097876.1   CTTAACAATA ATGGACCTGC ATCCAGGATC GGGAAAAACA AGAAGATACC

                ....|....| ....|....| ....|....| ....|....| ....|....|
                        5110       5120       5130       5140       5150
MZ291446.1   TACCAGCCAT AGTCCGTGAG GCCATAAAAA GGAAGCTGCG CACACTAATT
MG097876.1   TTCCGGCCAT AGTCCGTGAG GCCATAAAAA GGAAGCTGCG CACATTAATT

                ....|....| ....|....| ....|....| ....|....| ....|....|
                        5160       5170       5180       5190       5200
MZ291446.1   CTGGCTCCCA CAAGGGTTGT CGCCTCCGAA ATGGCAGAGG CGCTCAAGGG
MG097876.1   CTGGCCCCCA CAAGGGTTGT CGCTTCCGAA ATGGCAGAGG CGCTCAAGGG

                ....|....| ....|....| ....|....| ....|....| ....|....|
                        5210       5220       5230       5240       5250
MZ291446.1   AATGCCAATA AGGTACCAAA CAACAGCAGT GAAGAGTGAA CACACAGGAA
MG097876.1   AATGCCAATA AGGTACCAAA CAACAGCAGT GAAGAGTGAA CACACAGGAA

                ....|....| ....|....| ....|....| ....|....| ....|....|
                        5260       5270       5280       5290       5300
MZ291446.1   AAGAGATAGT TGACCTCATG TGTCACGCCA CCTTCACTAT GCGCCTCCTG
MG097876.1   AAGAGATAGT TGACCTCATG TGTCACGCCA CTTTCACCAT GCGCCTCCTG

                ....|....| ....|....| ....|....| ....|....| ....|....|
                        5310       5320       5330       5340       5350
MZ291446.1   TCTCCCGTGA GAGTTCCCAA TTATAACATG ATTATTATGG ATGAAGCACA
MG097876.1   TCTCCCGTGA GAGTTCCCAA TTACAACATG ATTATCATGG ATGAAGCACA

                ....|....| ....|....| ....|....| ....|....| ....|....|
                        5360       5370       5380       5390       5400
MZ291446.1   TTTCACCGAT CCATCCAGCA TAGCGGCTAG AGGGTACATC TCAACCCGAG
MG097876.1   TTTCACCGAT CCATCCAGCA TAGCGGCTAG AGGGTACATC TCAACCCGAG

                ....|....| ....|....| ....|....| ....|....| ....|....|
                        5410       5420       5430       5440       5450
MZ291446.1   TGGGCATGGG TGAAGCAGCT GCGATTTTCA TGACAGCCAC TCCCCCAGGA
MG097876.1   TGGGCATGGG TGAAGCAGCT GCGATCTTCA TGACAGCCAC TCCCCCAGGA
```

```
                ....|....| ....|....| ....|....| ....|....| ....|....|
                        5460       5470       5480       5490       5500
MZ291446.1      TCAGTGGAGG CCTTTCCACA GAGCAACGCA GTTATCCAAG ATGAGGAAAG
MG097876.1      TCAGTGGAGG CCTTTCCACA GAGCAACGCA GTTATCCAAG ATGAGGAAAG

                ....|....| ....|....| ....|....| ....|....| ....|....|
                        5510       5520       5530       5540       5550
MZ291446.1      AGATATTCCT GAGAGATCAT GGAACTCAGG TTATGAGTGG ATCACTGACT
MG097876.1      AGACATTCCT GAGAGATCAT GGAATTCAGG TTATGAGTGG ATCACTGACT

                ....|....| ....|....| ....|....| ....|....| ....|....|
                        5560       5570       5580       5590       5600
MZ291446.1      TCCCAGGCAA AACAGTTTGG TTTGTCCCAA GCATTAAATC AGGAAATGAT
MG097876.1      TCCCAGGCAA AACAGTTTGG TTTGTTCCAA GCATTAAATC AGGAAATGAT

                ....|....| ....|....| ....|....| ....|....| ....|....|
                        5610       5620       5630       5640       5650
MZ291446.1      ATTGCCAACT GCTTAAGAAA GAATGGGAAA CGAGTGATTC AATTGAGCAG
MG097876.1      ATTGCCAACT GCTTAAGAAA GAATGGGAAA CGGGTGATTC AATTGAGCAG

                ....|....| ....|....| ....|....| ....|....| ....|....|
                        5660       5670       5680       5690       5700
MZ291446.1      AAAAACCTTT GACACAGAGT ACCAAAAAAC AAAAAATAAC GACTGGGACT
MG097876.1      GAAAACCTTT GACACAGAGT ACCAAAAAAC AAAAAATAAC GACTGGGACT

                ....|....| ....|....| ....|....| ....|....| ....|....|
                        5710       5720       5730       5740       5750
MZ291446.1      ACGTCGTCAC AACAGACATC TCCGAAATGG GAGCAAATTT CCGGGCCGAC
MG097876.1      ACGTCGTCAC AACAGACATC TCCGAAATGG GAGCAAATTT CCGGGCCGAC

                ....|....| ....|....| ....|....| ....|....| ....|....|
                        5760       5770       5780       5790       5800
MZ291446.1      AGGGTGATAG ACCCAAGACG GTGTCTGAAA CCGGTAATAC TAAAAGATGG
MG097876.1      AGGGTGATAG ACCCAAGACG GTGTCTGAAA CCGGTAATAC TAAAAGATGG

                ....|....| ....|....| ....|....| ....|....| ....|....|
                        5810       5820       5830       5840       5850
MZ291446.1      TCCAGAGCGT GTCATCTTAG CAGGACCGAT GCCAGTGACT GTGGCTAGTG
MG097876.1      TCCAGAGCGT GTCATTTTAG CAGGACCGAT GCCAGTGACT GTGGCCAGTG

                ....|....| ....|....| ....|....| ....|....| ....|....|
                        5860       5870       5880       5890       5900
MZ291446.1      CCGCCCAAAG GAGAGGAAGA ATTGGAAGGA ACCACAACAA GGAAGGTGAT
MG097876.1      CCGCCCAGAG GAGAGGAAGA ATTGGAAGGA ACCACAATAA GGAAGGTGAT

                ....|....| ....|....| ....|....| ....|....| ....|....|
                        5910       5920       5930       5940       5950
MZ291446.1      CAGTACATCT ATATGGGACA GCCTTTAAAC AACGATGAAG ATCACGCTCA
MG097876.1      CAGTACATCT ATATGGGACA GCCTTTAAAC AACGATGAAG ATCACGCTCA

                ....|....| ....|....| ....|....| ....|....| ....|....|
                        5960       5970       5980       5990       6000
MZ291446.1      CTGGACGGAA GCAAAAATGC TCCTTGATAA TATAAACACA CCAGAAGGAA
MG097876.1      CTGGACGGAA GCAAAAATGC TACTTGATAA TATAAACACA CCAGAAGGAA

                ....|....| ....|....| ....|....| ....|....| ....|....|
                        6010       6020       6030       6040       6050
MZ291446.1      TTATCCCAGC CCTCTTCGAG CCGGAGAGAG AAAAGAGTGC GGCAATAGAC
MG097876.1      TTATCCCAGC TCTCTTCGAG CCAGAGAGAG AAAAGAGTGC GGCAATAGAC

                ....|....| ....|....| ....|....| ....|....| ....|....|
```

```
                   6060       6070       6080       6090       6100
MZ291446.1   GGGGAATACA GACTGCGGGG TGAAGCAAGG AAAACGTTTG TGGAGCTCAT
MG097876.1   GGGGAATACA GACTGCGAGG TGAAGCAAGG AAAACGTTTG TGGAGCTCAT

             ....|....| ....|....| ....|....| ....|....| ....|....|
                   6110       6120       6130       6140       6150
MZ291446.1   GAGAAGAGGA GATCTACCTG TCTGGCTATC TTACAAAGTT GCCTCAGAAG
MG097876.1   GAGAAGAGGA GATCTACCTG TATGGCTATC CTACAAAGTT GCCTCAGAAG

             ....|....| ....|....| ....|....| ....|....| ....|....|
                   6160       6170       6180       6190       6200
MZ291446.1   GCTTCCAATA CTCTGACAGA AGATGGTGCT TTGACGGGGA AAGGAACAAC
MG097876.1   GCTTCCAGTA CTCTGATAGA AGATGGTGCT TTGACGGGGA AAGGAACAAC

             ....|....| ....|....| ....|....| ....|....| ....|....|
                   6210       6220       6230       6240       6250
MZ291446.1   CAGGTGTTGG AAGAGAACAT GGACGTGGAG ATCTGGACAA AAGAAGGAGA
MG097876.1   CAGGTGTTGG AGGAGAACAT GGACGTGGAG ATCTGGACGA AAGAAGGAGA

             ....|....| ....|....| ....|....| ....|....| ....|....|
                   6260       6270       6280       6290       6300
MZ291446.1   AAGGAAGAAA CTACGACCCC GCTGGCTGGA CGCCAGAACA TACTCAGACC
MG097876.1   AAGAAAGAAA CTACGACCCC GCTGGCTGGA TGCCAGAACA TACTCAGACC

             ....|....| ....|....| ....|....| ....|....| ....|....|
                   6310       6320       6330       6340       6350
MZ291446.1   CACTGGCCCT GCGCGAGTTT AAAGAGTTTG CAGCAGGAAG AAGAAGCGTC
MG097876.1   CACTGGCCCT GCGCGAGTTT AAAGAGTTTG CAGCAGGAAG AAGAAGCGTC

             ....|....| ....|....| ....|....| ....|....| ....|....|
                   6360       6370       6380       6390       6400
MZ291446.1   TCAGGTGATC TAATATTGGA AATAGGGAAA CTTCCACAAC ACTTGACGCA
MG097876.1   TCAGGTGATC TAATATTGGA AATAGGGAAA CTTCCACAAC ACTTGACGCA

             ....|....| ....|....| ....|....| ....|....| ....|....|
                   6410       6420       6430       6440       6450
MZ291446.1   AAGGGCCCAG AATGCCTTGG ATAACCTGGT CATGTTGCAC AACTCCGAAC
MG097876.1   AAGGGCCCAG AATGCCTTGG ACAACCTGGT TATGTTGCAC AACTCCGAAC

             ....|....| ....|....| ....|....| ....|....| ....|....|
                   6460       6470       6480       6490       6500
MZ291446.1   AAGGAGGAAG AGCCTATAGA CATGCAATGG AAGAACTGCC AGACACCATA
MG097876.1   AAGGAGGAAG AGCCTATAGA CATGCAATGG AAGAACTGCC AGACACCATA

             ....|....| ....|....| ....|....| ....|....| ....|....|
                   6510       6520       6530       6540       6550
MZ291446.1   GAAACGTTAA TGCTCCTAGC TTTGATAGCT GTGTTAACTG GTGGAGTGAC
MG097876.1   GAAACGTTGA TGCTCCTAGC TTTGATAGCT GTGTTAACTG GTGGAATGAC

             ....|....| ....|....| ....|....| ....|....| ....|....|
                   6560       6570       6580       6590       6600
MZ291446.1   GCTGTTCTTC CTATCAGGAA GAGGCTTAGG GAAAACATCT ATCGGCCTAC
MG097876.1   GCTGTTCTTC CTATCAGGAA GGGGCTTAGG GAAAACATCT ATCGGCCTAC

             ....|....| ....|....| ....|....| ....|....| ....|....|
                   6610       6620       6630       6640       6650
MZ291446.1   TCTGCGTGAT GGCTTCAAGC GTATTGCTAT GGATGGCCAG TGTAGAGCCC
MG097876.1   TCTGCGTGAT GGCTTCAAGC GTACTGCTAT GGATGGCCAG TGTAGAGCCC

             ....|....| ....|....| ....|....| ....|....| ....|....|
                   6660       6670       6680       6690       6700
```

```
MZ291446.1   CATTGGATAG CCGCCTCCAT CATACTGGAG TTCTTCCTGA TGGTGCTGCT
MG097876.1   CATTGGATAG CGGCCTCCAT CATACTGGAA TTCTTCCTGA TGGTGCTGCT

             ....|....| ....|....| ....|....| ....|....| ....|....|
                   6710       6720       6730       6740       6750
MZ291446.1   CATTCCAGAG CCAGACAGAC AACGCACTCC GCAGGACAAT CAGCTGGCAT
MG097876.1   TATTCCAGAG CCAGACAGAC AACGCACTCC GCAGGACAAC CAGTTGGCAT

             ....|....| ....|....| ....|....| ....|....| ....|....|
                   6760       6770       6780       6790       6800
MZ291446.1   ATGTGGTGAT AGGTTTGTTA TTCATGATAC TAACAGTAGC AGCCAATGAG
MG097876.1   ATGTGGTGAT AGGTTTGTTA TTCATGATAC TGACAGTAGC AGCCAATGAG

             ....|....| ....|....| ....|....| ....|....| ....|....|
                   6810       6820       6830       6840       6850
MZ291446.1   ATGGGACTGC TGGAAACCAC AAAGAAAGAC TTAGGGATTG GCCATGTGGC
MG097876.1   ATGGGATTGC TGGAAACCAC AAAGAAAGAC TTAGGGATTG GCCATGTGGC

             ....|....| ....|....| ....|....| ....|....| ....|....|
                   6860       6870       6880       6890       6900
MZ291446.1   TGTTGAAAAC CACCACCATG CCACAATGCT GGACGTAGAC TTACATCCAG
MG097876.1   TGTTGAAAAT CACCACCATG CCACAATGCT GGACGTAGAC TTACATCCAG

             ....|....| ....|....| ....|....| ....|....| ....|....|
                   6910       6920       6930       6940       6950
MZ291446.1   CTTCAGCCTG GACCCTTTAT GCAGTGGCCA CAACAATCAT CACTCCCATG
MG097876.1   CTTCAGCCTG GACCCTCTAT GCAGTGGCCA CAACAATTAT CACTCCCATG

             ....|....| ....|....| ....|....| ....|....| ....|....|
                   6960       6970       6980       6990       7000
MZ291446.1   ATGAGGCACA CAATCGAAAA CACAACAGCA AACATTTCCC TGACAGCCAT
MG097876.1   ATGAGGCACA CAATCGAAAA CACAACAGCA AACATTTCCC TGACAGCCAT

             ....|....| ....|....| ....|....| ....|....| ....|....|
                   7010       7020       7030       7040       7050
MZ291446.1   TGCAAATCAG GCAGCTATAT TGATGGGACT TGACAAAGGA TGGCCAATAT
MG097876.1   TGCAAACCAG GCAGCTATAT TGATGGGACT TGACAAAGGA TGGCCAATAT

             ....|....| ....|....| ....|....| ....|....| ....|....|
                   7060       7070       7080       7090       7100
MZ291446.1   CGAAGATGGA CATAGGAGTT CCACTTCTCG CCTTGGGGTG CTATTCCCAG
MG097876.1   CGAAGATGGA CATAGGAGTT CCACTTCTCG CCTTGGGGTG CTATTCCCAG

             ....|....| ....|....| ....|....| ....|....| ....|....|
                   7110       7120       7130       7140       7150
MZ291446.1   GTGAATCCAC TGACGCTGAC AGCGGCGGTA TTGATGCTAG TGGCCCACTA
MG097876.1   GTGAATCCAC TGACGCTGAC AGCGGCGGTA TTGATGCTAG TGGCCCATTA

             ....|....| ....|....| ....|....| ....|....| ....|....|
                   7160       7170       7180       7190       7200
MZ291446.1   CGCTATAATT GGACCTGGAC TGCAAGCAAA AGCTACTAGA GAAGCTCAAA
MG097876.1   CGCTATAATT GGACCTGGAC TGCAAGCAAA AGCTACTAGA GAAGCTCAAA

             ....|....| ....|....| ....|....| ....|....| ....|....|
                   7210       7220       7230       7240       7250
MZ291446.1   AAAGGACAGC GGCCGGAATA ATGAAAAATC CAACTGTTGA TGGAATCGTT
MG097876.1   AAAGGACAGC GGCCGGAATA ATGAAAAATC CAACCGTTGA TGGAATCGTT

             ....|....| ....|....| ....|....| ....|....| ....|....|
                   7260       7270       7280       7290       7300
MZ291446.1   GCAATAGACC TGGACCCTGT GGTTTATGAT GCGAAATTTG AGAAACAACT
```

```
MG097876.1   GCAATAGATT TGGACCCTGT GGTTTATGAT GCGAAATTTG AGAAACAACT

             ....|....| ....|....| ....|....| ....|....| ....|....|
                   7310       7320       7330       7340       7350
MZ291446.1   AGGCCAAATA ATGTTGCTGA TACTATGCAC ATCACAGATT CTCTTGATGC
MG097876.1   AGGCCAAATA ATGCTGTTGA TACTATGCAC ATCACAGATC CTCTTGATGC

             ....|....| ....|....| ....|....| ....|....| ....|....|
                   7360       7370       7380       7390       7400
MZ291446.1   GGACTACATG GGCCTTGTGC GAATCCATCA CACTGGCCAC TGGACCTCTG
MG097876.1   GGACTACATG GGCCTTGTGC GAATCCATTA CACTGGCCAC TGGACCTCTG

             ....|....| ....|....| ....|....| ....|....| ....|....|
                   7410       7420       7430       7440       7450
MZ291446.1   ACCACGCTCT GGGAGGGATC TCCAGGAAAA TTTTGGAACA CCACGATAGC
MG097876.1   ACCACGCTCT GGGAGGGATC TCCAGGAAAA TTTTGGAACA CCACGATAGC

             ....|....| ....|....| ....|....| ....|....| ....|....|
                   7460       7470       7480       7490       7500
MZ291446.1   AGTTTCCATG GCAAACATTT TCAGAGGAAG TTATCTAGCA GGAGCAGGTC
MG097876.1   AGTTTCCATG GCAAACATTT TCAGAGGAAG TTATCTAGCA GGAGCAGGTC

             ....|....| ....|....| ....|....| ....|....| ....|....|
                   7510       7520       7530       7540       7550
MZ291446.1   TGGCTTTCTC ATTAATGAAA TCTCTAGGAG GAGGTAGGAG AGGCACGGGA
MG097876.1   TGGCCTTCTC ATTAATGAAA TCTCTAGGAG GAGGCAGGAG AGGCACGGGA

             ....|....| ....|....| ....|....| ....|....| ....|....|
                   7560       7570       7580       7590       7600
MZ291446.1   GCCCAAGGGG AAACACTGGG AGAGAAATGG AAAAGACAGC TGAACCAACT
MG097876.1   GCCCAAGGGG AAACACTGGG AGAGAAATGG AAAAGACAGC TGAACCAACT

             ....|....| ....|....| ....|....| ....|....| ....|....|
                   7610       7620       7630       7640       7650
MZ291446.1   GAGCAAGTCA GAATTCAACA CCTATAAAAG GAGTGGGATT ATGGAAGTGG
MG097876.1   GAGCAAGTCA GAATTCAACA CCTATAAAAG GAGTGGGATT ATGGAAGTGG

             ....|....| ....|....| ....|....| ....|....| ....|....|
                   7660       7670       7680       7690       7700
MZ291446.1   ACAGATCCGA AGCCAAAGAG GGACTGAAAA GAGGAGAAAC AACCAAACAT
MG097876.1   ACAGATCCGA AGCCAAAGAG GGACTGAAAA GAGGAGAAAC AACCAAACAT

             ....|....| ....|....| ....|....| ....|....| ....|....|
                   7710       7720       7730       7740       7750
MZ291446.1   GCAGTGTCGA GAGGAACCGC TAAATTGAGG TGGTTTGTGG AGAGGAACCT
MG097876.1   GCAGTGTCGA GAGGAACCGC TAAATTGAGG TGGTTTGTGG AGAGGAACCT

             ....|....| ....|....| ....|....| ....|....| ....|....|
                   7760       7770       7780       7790       7800
MZ291446.1   CGTGAAACCA GAAGGGAAAG TCATAGACCT CGGTTGTGGA AGAGGTGGCT
MG097876.1   TGTGAAACCA GAAGGGAAAG TCATAGACCT CGGTTGTGGA AGAGGTGGCT

             ....|....| ....|....| ....|....| ....|....| ....|....|
                   7810       7820       7830       7840       7850
MZ291446.1   GGTCATACTA TTGCGCTGGG CTGAAGAAAG TCACAGAGGT GAAGGGATAC
MG097876.1   GGTCATACTA TTGCGCTGGG CTGAAGAAAG TCACAGAAGT GAAGGGATAC

             ....|....| ....|....| ....|....| ....|....| ....|....|
                   7860       7870       7880       7890       7900
MZ291446.1   ACAAAAGGAG GACCTGGACA CGAAGAACCA ATCCCAATGG CGACCTATGG
MG097876.1   ACAAAAGGAG GACCTGGACA CGAAGAACCA ATCCCAATGG CGACCTATGG
```

```
               ....|....| ....|....| ....|....| ....|....| ....|....|
                       7910       7920       7930       7940       7950
MZ291446.1     ATGGAACCTA GTAAAGCTGT ACTCCGGGAA AGACGTGTTC TTTATACCAC
MG097876.1     ATGGAACCTA GTAAAGCTGT ACTCCGGGAA AGATGTATTC TTTATGCCAC

               ....|....| ....|....| ....|....| ....|....| ....|....|
                       7960       7970       7980       7990       8000
MZ291446.1     CTGAGAAATG TGACACCCTC TTGTGTGATA TTGGTGAGTC CTCTCCAAAC
MG097876.1     ATGAGAAGTG TGACACCCTT TTATGTGATA TTGGTGAGTC CTCTCCAAAC

               ....|....| ....|....| ....|....| ....|....| ....|....|
                       8010       8020       8030       8040       8050
MZ291446.1     CCAACTATAG AGGAAGGAAG AACGCTACGC GTCCTAAAGA TGGTGGAACC
MG097876.1     CCAACTATAG AGGAAGGAAG AACGCTACGC GTCCTAAAGA TGGTGGAACC

               ....|....| ....|....| ....|....| ....|....| ....|....|
                       8060       8070       8080       8090       8100
MZ291446.1     ATGGCTCAGA GGAAACCAAT TTTGCATAAA AATTCTGAAT CCCTACATGC
MG097876.1     ATGGCTCAGA GGAAACCAAT TTTGCATAAA AGTTCTGAAT CCCTACATGC

               ....|....| ....|....| ....|....| ....|....| ....|....|
                       8110       8120       8130       8140       8150
MZ291446.1     CAAGTGTGGT GGAAACTCTG GAGCAAATGC AAAGAAAACA CGGAGGAATG
MG097876.1     CAAGTGTGGT GGAAACTCTG GAGCAAATGC AGAGAAAACA TGGAGGAATG

               ....|....| ....|....| ....|....| ....|....| ....|....|
                       8160       8170       8180       8190       8200
MZ291446.1     CTGGTGCGAA ATCCACTTTC AAGGAATTCC ACTCATGAAA TGTATTGGGT
MG097876.1     CTAGTGCGAA ATCCACTTTC AAGAAATTCT ACTCATGAAA TGTATTGGGT

               ....|....| ....|....| ....|....| ....|....| ....|....|
                       8210       8220       8230       8240       8250
MZ291446.1     TTCATGTGGA ACAGGAAACA TTGTGTCAGC AGTAAACATG ACATCCAGAA
MG097876.1     TTCATGTGGA ACAGGAAACA TTGTGTCAGC AGTAAACATG ACATCCAGAA

               ....|....| ....|....| ....|....| ....|....| ....|....|
                       8260       8270       8280       8290       8300
MZ291446.1     TGTTGCTAAA TCGGTTCACA ATGGCTCATA GGAAACCAAC ATACGAAAGA
MG097876.1     TGTTGCTAAA TCGATTCACA ATGGCTCATA GGAAACCAAC ATATGAAAGA

               ....|....| ....|....| ....|....| ....|....| ....|....|
                       8310       8320       8330       8340       8350
MZ291446.1     GACGTGGACC TAGGCGCCGG AACAAGACAT GTGGCAGTAG AACCAGAGGT
MG097876.1     GACGTGGACC TAGGCGCCGG AACAAGACAT GTGGCAGTGG AACCAGAGGT

               ....|....| ....|....| ....|....| ....|....| ....|....|
                       8360       8370       8380       8390       8400
MZ291446.1     AGCTAACCTA GATATCATTG GCCAGAGGAT AGAGAACATA AAACATGAAC
MG097876.1     AGCCAACCTA GATATTATTG GCCAGAGGAT AGAGAACATA AAACATGAAC

               ....|....| ....|....| ....|....| ....|....| ....|....|
                       8410       8420       8430       8440       8450
MZ291446.1     ACAAGTCAAC ATGGCATTAT GATGAGGACA ATCCATATAA AACATGGGCC
MG097876.1     ACAAGTCAAC ATGGCATTAT GATGAGGACA ATCCATATAA AACATGGGCC

               ....|....| ....|....| ....|....| ....|....| ....|....|
                       8460       8470       8480       8490       8500
MZ291446.1     TATCATGGAT CATATGAGGT CAAACCATCA GGATCAGCCT CATCCATGGT
MG097876.1     TATCATGGAT CATATGAGGT CAAGCCATCA GGATCAGCCT CATCCATGGT
```

```
                ....|....| ....|....| ....|....| ....|....| ....|....|
                        8510       8520       8530       8540       8550
MZ291446.1      CAATGGTGTG GTGAAACTGC TCACCAAACC ATGGGATGTC ATCCCCATGG
MG097876.1      CAATGGTGTA GTGAAACTGC TCACCAAACC ATGGGATGTC ATCCCCATGG

                ....|....| ....|....| ....|....| ....|....| ....|....|
                        8560       8570       8580       8590       8600
MZ291446.1      TCACACAAAT AGCTATGACT GACACTACAC CCTTTGGACA ACAGAGGGTA
MG097876.1      TCACACAAAT AGCCATGACT GACACTACAC CCTTTGGACA ACAGAGGGTG

                ....|....| ....|....| ....|....| ....|....| ....|....|
                        8610       8620       8630       8640       8650
MZ291446.1      TTTAAAGAGA AAGTTGACAC ACGCACACCA AAAGCAAAAC GAGGCACAGC
MG097876.1      TTTAAAGAGA AAGTTGACAC ACGCACACCA AAAGCAAAAC GAGGCACAGC

                ....|....| ....|....| ....|....| ....|....| ....|....|
                        8660       8670       8680       8690       8700
MZ291446.1      ACAAATCATG GAGGTGACAG CCAGGTGGTT ATGGGGTTTT CTTTCTAGAA
MG097876.1      ACAAATCATG GAGGTGACAG CCAGGTGGTT ATGGGGTTTT CTTTCTAGAA

                ....|....| ....|....| ....|....| ....|....| ....|....|
                        8710       8720       8730       8740       8750
MZ291446.1      ACAAGAAGCC AAGAATTTGT ACAAGAGAGG AGTTCACAAG AAAAGTTAGG
MG097876.1      ACAAGAAGCC AAGAATTTGT ACAAGAGAGG AGTTCACAAG AAAAGTTAGG

                ....|....| ....|....| ....|....| ....|....| ....|....|
                        8760       8770       8780       8790       8800
MZ291446.1      TCAAACGCAG CCATCGGAGC AGTGTTCATT GACGAAAACC AATGGAACTC
MG097876.1      TCAAACGCAG CCATCGGAGC AGTGTTCATT GACGAAAATC AATGGAACTC

                ....|....| ....|....| ....|....| ....|....| ....|....|
                        8810       8820       8830       8840       8850
MZ291446.1      AGCAAAAGAA GCAGTGGAAG ATGAGCGGTT CTGGGACCTT GTGCACAGAG
MG097876.1      AGCAAAAGAA GCAGTGGAAG ATGAGCGGTT CTGGGACCTT GTGCACAGAG

                ....|....| ....|....| ....|....| ....|....| ....|....|
                        8860       8870       8880       8890       8900
MZ291446.1      AGAGGGAGCT TCACAAACAG GGAAAATGTG CCACGTGTGT TTACAATATG
MG097876.1      AGAGGGAGCT TCACAAACAG GGAAAATGTG CCACGTGTGT TTACAACATG

                ....|....| ....|....| ....|....| ....|....| ....|....|
                        8910       8920       8930       8940       8950
MZ291446.1      ATGGGGAAGA GAGAGAAAAA GCTAGGAGAA TTTGGAAAGG CAAAAGGAAG
MG097876.1      ATGGGGAAGA GAGAGAAAAA GCTAGGAGAA TTTGGAAAGG CAAAAGGAAG

                ....|....| ....|....| ....|....| ....|....| ....|....|
                        8960       8970       8980       8990       9000
MZ291446.1      TCGTGCAATA TGGTACATGT GGTTGGGAGC ACGCTTTCTA GAGTTCGAAG
MG097876.1      TCGTGCAATA TGGTACATGT GGTTGGGAGC ACGCTTTCTA GAGTTCGAAG

                ....|....| ....|....| ....|....| ....|....| ....|....|
                        9010       9020       9030       9040       9050
MZ291446.1      CTCTTGGTTT CATGAACGAA GACCACTGGT TCAGCAGAGA GAATTCACTC
MG097876.1      CTCTTGGTTT CATGAACGAA GACCACTGGT TCAGCAGAGA GAATTCACTC

                ....|....| ....|....| ....|....| ....|....| ....|....|
                        9060       9070       9080       9090       9100
MZ291446.1      AGCGGAGTGG AAGGAGAAGG ACTCCACAAA CTTGGATATA TACTCAGAGA
MG097876.1      AGCGGAGTGG AAGGAGAAGG ACTCCACAAA CTTGGATATA TACTCAGAGA
```

```
             ....|....| ....|....| ....|....| ....|....| ....|....|
                  9110       9120       9130       9140       9150
MZ291446.1   CATATCAAAG ATTCCAGGGG GAAACATGTA CGCTGATGAC ACAGCCGGAT
MG097876.1   CATATCAAAG ATTTCAGGGG GAAACATGTA TGCTGATGAC ACAGCCGGAT

             ....|....| ....|....| ....|....| ....|....| ....|....|
                  9160       9170       9180       9190       9200
MZ291446.1   GGGATACAAG GATAACAGAG GATGACCTTC AGAATGAGGC CAAAATTACT
MG097876.1   GGGATACAAG GATAACAGAG GATGACCTTC AGAATGAGGC CAAAATTACT

             ....|....| ....|....| ....|....| ....|....| ....|....|
                  9210       9220       9230       9240       9250
MZ291446.1   GACATCATGG AACCCGAACA TGCCCTACTG GCTAAGGCAA TCTTCAAGCT
MG097876.1   GACATCATGG AACCCGAACA TGCTCTACTA GCTAAGGCAA TCTTCAAGCT

             ....|....| ....|....| ....|....| ....|....| ....|....|
                  9260       9270       9280       9290       9300
MZ291446.1   AACCTACCAA AATAAAGTGG TAAGGGTACA GAGACCAGCA AAAAATGGAA
MG097876.1   AACCTACCAA AATAAAGTGG TAAGGGTACA GAGACCAGCA AAAAATGGAA

             ....|....| ....|....| ....|....| ....|....| ....|....|
                  9310       9320       9330       9340       9350
MZ291446.1   CCGTGATGGA TGTCATATCC AGACGTGACC AGAGAGGAAG CGGCCAGGTC
MG097876.1   CTGTGATGGA TGTCATTTCC AGACGTGACC AGAGAGGAAG TGGCCAGGTC

             ....|....| ....|....| ....|....| ....|....| ....|....|
                  9360       9370       9380       9390       9400
MZ291446.1   GGAACTTATG GCTTAAACAC TTTCACCAAC ATGGAAGCCC AGCTGATAAG
MG097876.1   GGAACTTATG GCTTAAACAC TTTCACCAAC ATGGAAGCCC AGCTGATAAG

             ....|....| ....|....| ....|....| ....|....| ....|....|
                  9410       9420       9430       9440       9450
MZ291446.1   ACAAATGGAG TCTGAGGGAA TCTTTTCACC CAGTGAATTA GAGACCCCAA
MG097876.1   ACAAATGGAG TCTGAGGGAA TCTTTTCACC TAGTGAATTA GAGACCCCAA

             ....|....| ....|....| ....|....| ....|....| ....|....|
                  9460       9470       9480       9490       9500
MZ291446.1   ATTTAGCCGA GAGAGTTCTC GACTGGTTGG AAAAATATGG CGTCGAAAGG
MG097876.1   ATTTGGCCGA GAGAGTTCTC GACTGGCTGG AAAAATATGG CGTCGAAAGG

             ....|....| ....|....| ....|....| ....|....| ....|....|
                  9510       9520       9530       9540       9550
MZ291446.1   CTAAAAAGAA TGGCAATCAG TGGAGATGAC TGCGTAGTGA AACCAATTGA
MG097876.1   CTAAAAAGAA TGGCAATCAG TGGAGATGAC TGCGTAGTGA AACCAATTGA

             ....|....| ....|....| ....|....| ....|....| ....|....|
                  9560       9570       9580       9590       9600
MZ291446.1   TGATAGGTTT GCAACAGCCT TAACAGCTCT GAATGATATG GGAAAAGTAA
MG097876.1   TGATAGGTTT GCAACAGCCT TAACAGCTCT GAATGATATG GGAAAAGTAA

             ....|....| ....|....| ....|....| ....|....| ....|....|
                  9610       9620       9630       9640       9650
MZ291446.1   GAAAAGATAT ACCACAATGG GAACCCTCAA AAGGATGGAA TGATTGGCAA
MG097876.1   GAAAAGATAT ACCACAATGG GAACCCTCAA AAGGATGGAA TGATTGGCAA

             ....|....| ....|....| ....|....| ....|....| ....|....|
                  9660       9670       9680       9690       9700
MZ291446.1   CAAGTGCCTT TTTGTTCACA CCACTTCCAC CAGCTGATTA TGAAGGATGG
MG097876.1   CAAGTGCCTT TTTGTTCACA CCACTTCCAC CAGCTGATTA TGAAGGATGG

             ....|....| ....|....| ....|....| ....|....| ....|....|
```

```
                   9710      9720      9730      9740      9750
MZ291446.1   GAGGGAAATA GTGGTGCCAT GCCGCAACCA AGATGAACTT GTGGGTAGGG
MG097876.1   GAGGGAAATA GTGGTGCCAT GCCGCAACCA AGATGAACTT GTGGGTAGGG


             ....|....| ....|....| ....|....| ....|....| ....|....|
                   9760      9770      9780      9790      9800
MZ291446.1   CTCGAGTATC ACAAGGTGCT GGATGGAGCC TGAGAGAAAC CGCATGCCTA
MG097876.1   CTAGAGTATC ACAAGGTGCT GGATGGAGCC TGAGAGAAAC CGCATGCCTA


             ....|....| ....|....| ....|....| ....|....| ....|....|
                   9810      9820      9830      9840      9850
MZ291446.1   GGCAAATCAT ATGCACAAAT GTGGCAGCTG ATGTACTTCC ACAGGAGAGA
MG097876.1   GGCAAATCAT ATGCACAAAT GTGGCAGCTG ATGTACTTCC ACAGGAGAGA


             ....|....| ....|....| ....|....| ....|....| ....|....|
                   9860      9870      9880      9890      9900
MZ291446.1   CCTGAGACTA GCTGCTAACG CCATCTGTTC AGCCGTTCCA GTTGATTGGG
MG097876.1   CCTGAGACTA GCTGCTAATG CTATCTGTTC AGCCGTTCCA GTTGATTGGG


             ....|....| ....|....| ....|....| ....|....| ....|....|
                   9910      9920      9930      9940      9950
MZ291446.1   TCCCAACTAG CCGCACCACC TGGTCTATCC ATGCCCATCA CCAATGGATG
MG097876.1   TCCCAACTAG CCGCACCACC TGGTCGATCC ATGCCCATCA CCAATGGATG


             ....|....| ....|....| ....|....| ....|....| ....|....|
                   9960      9970      9980      9990     10000
MZ291446.1   ACAACAGAAG ACATGCTGTC AGTGTGGAAT AGAGTTTGGA TAGAGGAAAA
MG097876.1   ACAACAGAAG ACATGCTGTC AGTGTGGAAT AGGGTTTGGA TAGAGGAAAA


             ....|....| ....|....| ....|....| ....|....| ....|....|
                  10010     10020     10030     10040     10050
MZ291446.1   CCCATGGATG GAGGACAAAA CCCATATATC CAGTTGGGAA GATGTTCCAT
MG097876.1   CCCATGGATG GAGGACAAAA CCCATATATC CAGTTGGGAA GATGTTCCAT


             ....|....| ....|....| ....|....| ....|....| ....|....|
                  10060     10070     10080     10090     10100
MZ291446.1   ATTTAGGGAA AAGGGAAGAT CAATGGTGTG GATCCCTGAT AGGCTTAACA
MG097876.1   ATTTAGGGAA AAGGGAAGAT CAATGGTGTG GATCCCTGAT AGGCTTAACA


             ....|....| ....|....| ....|....| ....|....| ....|....|
                  10110     10120     10130     10140     10150
MZ291446.1   GCAAGGGCCA CCTGGGCCAC CAACATACAA GTGGCCATAA ACCAGGTGAG
MG097876.1   GCAAGGGCCA CCTGGGCCAC CAACATACAA GTGGCCATAA ACCAAGTGAG


             ....|....| ....|....| ....|....| ....|....| ....|....|
                  10160     10170     10180     10190     10200
MZ291446.1   AAGATTAATT GGGAATGAGA ATTATCTAGA TTACATGACA TCAATGAAGA
MG097876.1   AAGACTAATT GGGAATGAGA ATTATCTAGA TTACATGACA TCAATGAAGA


             ....|....| ....|....| ....|....| ....|....| ....|....|
                  10210     10220     10230     10240     10250
MZ291446.1   GATTCAAGAA CGAGAGTGAT CCCGAAGGGG CACTCTGGTG AGCCAACACA
MG097876.1   GATTCAAGAA CGAGAGTGAT CCCGAAGGGG CACTCTGGTG AGCCAACACA


             ....|....| ....|....| ....|....| ....|....| ....|....|
                  10260     10270     10280     10290     10300
MZ291446.1   TTTACAAAAT AAAGGAAAAT AAGAAATCAA ACAAGGCAAG AAGTCAGGCC
MG097876.1   TTTACAAAAT AAAGGAAAAT AAGAAATCAA ACAAGGCAAG AAGTCAGGCC


             ....|....| ....|....| ....|....| ....|....| ....|....|
                  10310     10320     10330     10340     10350
```

```
MZ291446.1   TGGATTAAGC CATAGTACGG TAAGAGCTAT GCTGCCTGTG AGCCCCGTCT
MG097876.1   GGATTAAGCC ATAGTACGGT AAGAGCTATG CTGCCTGTGA GCCCCGTCTA


             ....|....| ....|....| ....|....| ....|....| ....|....|
                  10360      10370      10380      10390      10400
MZ291446.1   AAGGACGTAA AAAGAAGTCA GGCCGAAAGC CACGGTTTGA GCAAACCGTG
MG097876.1   AGGACGTAAA ATGAAGTCAG GCCGAAAGCC ACGGTTTGAG CAAACCGTGC


             ....|....| ....|....| ....|....| ....|....| ....|....|
                  10410      10420      10430      10440      10450
MZ291446.1   CTGCCTGTAG CTCCATCGTG GGGATGTAAA AACCTGGGAG GCTGCAACCC
MG097876.1   TGCCTGTAGC TCCATCGTGG GGATGTAAAA ACCTGGGAGG CTGCAACCCA


             ....|....| ....|....| ....|....| ....|....| ....|....|
                  10460      10470      10480      10490      10500
MZ291446.1   ATGGAAGCTG TACGCATGGG GTAGCAGACT AGTGGTTAGA GGAGACCCCT
MG097876.1   TGGAAGCTGT ACGCATGGGG TAGCAGACTA GTGGTTAGAG GAGACCCCTC


             ....|....| ....|....| ....|....| ....|....| ....|....|
                  10510      10520      10530      10540      10550
MZ291446.1   CCCGAAACAC AACGCAGCAG CGGGGCCCAA CACCAGGGGA AGCTGTACCC
MG097876.1   CCGAAACACA ACGCAGCAGC GGGGCCCAAC ACCAGGGGAA GCTGTACCCT


             ....|....| ....|....| ....|....| ....|....| ....|....|
                  10560      10570      10580      10590      10600
MZ291446.1   TGGTGGTAAG GACTAGAGGT TAGAGGAGAC CCCCCGCATA ACAATAAACA
MG097876.1   GGTGGTAAGG ACTAGAGGTT AGAGGAGACC CCCCGCATAA CAATAAACAG


             ....|....| ....|..
                  10610
MZ291446.1   GCATATTGAC GCTGGGA
MG097876.1   CATATTGACG CTGGGA.
```
